# Supplementary material for: Comparison of Local Metabolic Changes in Diabetic Rodent Kidneys Using Mass Spectrometry Imaging
Source: Metabolites. 2023 Feb 22;13(3):324. doi: 10.3390/metabo13030324 (PMC10060001; doi:10.3390/metabo13030324)
Supplement: Supplementary file 1 [file metabolites-13-00324-s001.zip › metabolites-2198058-supplementary.pdf]

**Comparison of region-specific metabolic changes in kidney of HFD/STZ-induced diabetic rats and db/db mice based on air-flow-assisted desorption electrospray ionization-mass spectrometry imaging**

Xin Zhang[1,2], Yanhua Liu[1,2], Shu Yang[1,3], Xin Gao[1,2], Shuo Wang[1,2], Zhaoying Wang[1,2], Chen Zhang[1,2], Zhi Zhou[1,2], Yanhua Chen[1,2], Zhonghua Wang[1,2,*], and Zeper Abliz[1,2,3]

[1]Key Laboratory of Mass Spectrometry Imaging and Metabolomics (Minzu University of China), National Ethnic Affairs Commission, Beijing 100081, China

[2]Center for Imaging and Systems Biology, College of Life and Environmental Sciences, Minzu University of China, 27 Zhongguancun South Avenue, Beijing 100081, China

[3]Key Laboratory of Ethnomedicine of Ministry of Education, School of Pharmacy, Minzu University of China, Beijing 100081, P. R. China

[*]*Corresponding author*: Tel.: +86 10-68933506; e-mail: wangzhonghua@muc.edu.cn

# SUPPORTING INFORMATION

**Table of Contents**

**Tables and Figures**

**Table S1**. AFADESI-MSI parameter........................................................ **4**

**Table S2**. Discriminating metabolites obtained by negative AFADESI-MSI analysis of renal sections from control and HFD/STZ-induced diabetic nephropathy (DN) group.................................................................................................................... **4**

**Table S3**. Discriminating metabolites obtained by positive AFADESI-MSI analysis of renal sections from control and HFD/STZ-induced diabetic nephropathy (DN) group.................................................................................................................... **6**

**Table S4**. Discriminating metabolites obtained by negative AFADESI-MSI analysis of renal sections from control and db/db diabetic nephropathy (DN) group........................**6**

**Table S5**. Discriminating metabolites obtained by positive AFADESI-MSI analysis of renal sections from control and db/db diabetic nephropathy (DN) group........................**7**

**Figure S1.** H&E staining in the HFD/STZ-induced diabetic rats (**A, B**) and db/db mice (**C, D**) ................................................................................................................. **8**

**Figure S2.** Fold change plots of discriminating metabolites in HFD/STZ-induced diabetic rats versus db/db mice..........................................................................**9**

**Figure S3.** The comparison of discriminating metabolites associated DN between the two models.............................................................................................................. **10**

**Figure S4** Regulation of discriminating metabolites in HFD/STZ-induced diabetic rats and db/db mice................................................. .................................................. **11**

**Figure S5.** Fan chart of discriminating lipid species in HFD/STZ-induced diabetic rats and db/db mice........................................................................................................ **11**

**Figure S6.** Histogram of metabolites involved in lipid metabolism in HFD/STZ-induced diabetic rats........................................................................................................ **12**

**Figure S7.** Histogram of metabolites involved in lipid metabolism in db/db mice.......**14**

**Figure S8.** Histogram of metabolites involved in glycolysis and TCA cycle in HFD/STZ-induced diabetic rats (A) and db/db mice(B) ............................................**15**

**Figure S9.** Histogram of metabolites involved in purine metabolism in HFD/STZ-induced diabetic rats (A) and db/db mice(B) ............................................................**15**

**Figure S10.** Histogram of metabolites involved in carnitine and choline metabolism in HFD/STZ-induced diabetic rats (A) and db/db mice(B) ............................................**16**

**Supplementary Methods**

**1. LC-MS/MS analysis** ........................................................................................**16**

## Supporting Information –Tables and Figures

**Table S1**. AFADESI-MSI parameter

| AFADESI-MSI | Value |
|---|---|
| Spray voltage | ±5KV |
| Transmission tube voltage | ±3KV |
| Solvent composition | CAN/$H_2O$ (8:2v/v) |
| Spray solvent flow rate | 7uL/min; 5uL/min |
| Nebulizer pressure | 0.6MPa; 0.5MPa |
| X-axis scan speed | 0.2mm/s |
| Y-axis inter-row distance | 0.2mm |
| Z vertical distance | 0 |
| Delay start time | 6s |
| Scan range | 100-1000 Da |
| Mass resolution | 120,000 |
| Capillary temperature | 350 ℃ |

**Table S2**. Discriminating metabolites obtained by negative AFADESI-MSI analysis of renal sections from control and HFD/STZ-induced diabetic nephropathy (DN) group.

| Species | Metabolite identification | Elemental composition | Adduct | Theoretical *m/z* | Measured *m/z* | Delta (ppm) | MS/MS | Distribution | FC(W) |
|---|---|---|---|---|---|---|---|---|---|
| Amino acid | Citrate | $C_6H_8O_7$ | $[M-H]^-$ | 191.0197 | 191.0204 | 3.72 | 129, 173, 191 | OM | 0.86 |
| Amino- acid | Aspartate | $C_4H_7NO_4$ | $[M-H]^-$ | 132.0302 | 132.0300 | -1.36 | 71, 88, 115, 132 | W, OM, IM | 0.67 |
| Amino- acid | Glutamate | $C_5H_9NO_4$ | $[M-H]^-$ | 146.0459 | 146.0457 | -1.64 | 102, 128, 146 | OM | 0.81 |
| Amino acid | Histidylaspartic acid | $C_{10}H_{14}N_4O_5$ | $[M-H]^-$ | 269.0891 | 269.0887 | -1.52 | 269 | C | 2.27 |
| DG | DG (38:3) (OH) | $C_{41}H_{74}O_6$ | $[M-H]^-$ | 661.5413 | 661.5425 | 1.84 | 661 | OM | 1.28 |
| FAHFA | FAHFA (34:0) | $C_{34}H_{66}O_4$ | $[M-H]^-$ | 537.4888 | 537.4896 | 1.44 | 255, 537 | W, C, OM | 2.70 |
| FAHFA | FAHFA (34:1) | $C_{34}H_{64}O_4$ | $[M-H]^-$ | 535.4732 | 535.4741 | 1.60 | 253, 535 | IM | 0.94 |
| Fatty acid | Linolenic acid | $C_{18}H_{30}O_2$ | $[M-H]^-$ | 277.2173 | 277.2178 | 1.70 | 59, 277 | W, C, OM, IM | 0.52 |
| Fatty acid | Linoleic acid | $C_{18}H_{32}O_2$ | $[M-H]^-$ | 279.233 | 279.2334 | 1.40 | 59, 26, 1279 | W, C, OM, IM | 0.68 |
| Fatty acid | Oleic acid | $C_{18}H_{34}O_2$ | $[M-H]^-$ | 281.2486 | 281.2489 | 1.11 | 59, 237, 281 | W, C, OM | 2.24 |
| Fatty acid | Eicosapentaenoic acid | $C_{20}H_{30}O_2$ | $[M-H]^-$ | 301.2173 | 301.2184 | 3.69 | 257, 283, 301 | W, C, OM, IM | 0.48 |
| Fatty acid | Eicosenoic acid | $C_{20}H_{38}O_2$ | $[M-H]^-$ | 309.2799 | 309.2802 | 0.95 | 265, 309 | W, C, OM, IM | 1.99 |
| Fatty acid | Docosahexaenoic acid | $C_{22}H_{32}O_2$ | $[M-H]^-$ | 327.2330 | 327.2328 | -0.70 | 67,121, 229, 283, 309, 327 | W, C, OM, IM | 0.47 |
| Fatty acid | Nervonic acid | $C_{24}H_{46}O_2$ | $[M-H]^-$ | 365.3425 | 365.3430 | 1.24 | 365 | W, C, OM, IM | 2.19 |
| LysoPE | LysoPE (16:0) | $C_{21}H_{44}NO_7P$ | $[M-H]^-$ | 452.2783 | 452.2799 | 3.59 | 214, 255, 253, 452 | W, C, OM | 0.48 |
| LysoPE | LysoPE (20:4) | $C_{25}H_{44}NO_7P$ | $[M-H]^-$ | 500.2783 | 500.2786 | 0.61 | 140, 214, 259, 303, 439, 500 | W, C, OM | 0.63 |
| LysoPG | LysoPG (18:1) | $C_{24}H_{47}O_9P$ | $[M-H]^-$ | 509.2885 | 509.2896 | 2.15 | 152, 281,509 | W, C, OM | 3.97 |

| | | | | | | | | | |
|---|---|---|---|---|---|---|---|---|---|
| LysoPI | LysoPI (16:0) | $C_{25}H_{49}O_{12}P$ | $[M-H]^-$ | 571.2889 | 571.2913 | 4.22 | 152, 255, 241, 391, 571 | C | 0.58 |
| Nucleoside& Nucleotide& Nitrogenbase | AMP | $C_{10}H_{14}N_5O_7P$ | $[M-H]^-$ | 346.0558 | 346.0566 | 2.20 | 78, 96, 134, 211, 346 | OM | 0.89 |
| Nucleoside& Nucleotide& Nitrogenbase | Inosine | $C_{10}H_{12}N_4O_5$ | $[M-H]^-$ | 267.0735 | 267.0746 | 4.01 | 107, 267 | W, C, IM | 0.54 |
| Organic-acid | 2(R)-hydroxydocosanoic acid | $C_{22}H_{44}O_3$ | $[M-H]^-$ | 355.3218 | 355.3221 | 0.71 | 309, 355 | OM | 3.88 |
| Organic-acid | Taurine | $C_2H_7NO_3S$ | $[M-H]^-$ | 124.0074 | 124.0073 | -0.48 | 80, 124 | W, C, OM, IM | 0.58 |
| PA | PA (34:2) | $C_{37}H_{69}O_8P$ | $[M-H]^-$ | 671.4657 | 671.4663 | 0.92 | 671 | W, C, OM | 0.41 |
| PE | PE (34:1) | $C_{39}H_{76}NO_8P$ | $[M-H]^-$ | 716.5236 | 716.5250 | 1.91 | 716 | W, C, OM | 0.52 |
| PE | PE (36:2) | $C_{41}H_{78}NO_8P$ | $[M-H]^-$ | 742.5392 | 742.5402 | 1.38 | 253, 742 | W, C | 0.57 |
| PE | PE (36:4) | $C_{41}H_{74}NO_8P$ | $[M-H]^-$ | 738.5079 | 738.5093 | 1.88 | 227, 738 | W, C, OM | 0.56 |
| PE | PE (38:4) | $C_{43}H_{78}NO_8P$ | $[M-H]^-$ | 766.539 | 766.5401 | 1.39 | 196, 281, 766 | W, C, OM | 0.66 |
| PE | PE(p-38:6) | $C_{43}H_{74}NO_7P$ | $[M-H]^-$ | 746.513 | 746.5113 | -2.32 | 746 | W, C, OM, IM | 0.31 |
| PE | PE (38:6) | $C_{43}H_{74}NO_8P$ | $[M-H]^-$ | 762.5079 | 762.5087 | 1.08 | 484, 762 | W, C, OM, IM | 0.33 |
| PE | Phosphoryle thanolamine | $C_2H_8NO_4P$ | $[M-H]^-$ | 140.0118 | 140.0117 | -0.71 | 78, 140 | OM | 0.78 |
| PG | PG (32:0) | $C_{38}H_{75}O_{10}P$ | $[M-H]^-$ | 721.5025 | 721.5026 | 0.16 | 255, 721 | W, C | 0.46 |
| PG | PG (34:2) | $C_{40}H_{75}O_{10}P$ | $[M-H]^-$ | 745.5025 | 745.5036 | 1.42 | 255, 279, 745 | C | 0.69 |
| PG | PG (36:2) | $C_{42}H_{79}O_{10}P$ | $[M-H]^-$ | 773.5338 | 773.5347 | 1.21 | 279, 283, 773 | W, C, OM | 2.52 |
| PG | PG (36:3) | $C_{42}H_{77}O_{10}P$ | $[M-H]^-$ | 771.5182 | 771.5189 | 0.93 | 283, 771 | W, C, OM | 3.36 |
| PG | PG (36:4) | $C_{42}H_{75}O_{10}P$ | $[M-H]^-$ | 769.5025 | 769.5051 | 3.37 | 255, 303, 769 | C | 0.79 |
| PG | PG (40:6) | $C_{46}H_{79}O_{10}P$ | $[M-H]^-$ | 821.5338 | 821.5354 | 1.97 | 303, 821 | W, C, OM | 3.04 |
| PG | PG (40:8) | $C_{46}H_{75}O_{10}P$ | $[M-H]^-$ | 817.5025 | 817.5037 | 1.44 | 303, 817 | W, C, OM, IM | 0.43 |
| PG | PG (42:10) | $C_{48}H_{75}O_{10}P$ | $[M-H]^-$ | 841.5025 | 841.5035 | 1.21 | 303, 841 | W, OM, IM | 0.53 |
| PG | PG (44:12) | $C_{50}H_{75}O_{10}P$ | $[M-H]^-$ | 865.5025 | 865.5036 | 1.22 | 327, 865 | W, C, OM, IM | 0.22 |
| PI | PI (34:2) | $C_{43}H_{79}O_{13}P$ | $[M-H]^-$ | 833.5186 | 833.5190 | 0.52 | 279, 833 | W, C, OM | 0.35 |
| PI | PI (18:1(9Z)/18:1(9Z)) | $C_{45}H_{83}O_{13}P$ | $[M-H]^-$ | 861.5499 | 861.5511 | 1.41 | 281, 861 | W, C, OM | 0.51 |
| PI | PI (36:4) | $C_{45}H_{79}O_{13}P$ | $[M-H]^-$ | 857.5186 | 857.5193 | 0.79 | 303, 391, 553, 857 | W, C, OM | 0.50 |
| PI | PI (38:5) | $C_{47}H_{81}O_{13}P$ | $[M-H]^-$ | 883.5342 | 883.5350 | 0.95 | 303, 417, 883 | C | 0.66 |
| PI | PI (40:6) | $C_{49}H_{83}O_{13}P$ | $[M-H]^-$ | 909.5499 | 909.5499 | -0.01 | 283, 909 | W, C, OM, IM | 0.33 |
| PS | PS (34:1) | $C_{40}H_{76}NO_{10}P$ | $[M-H]^-$ | 760.5134 | 760.5139 | 0.66 | 227, 760 | W, C, OM | 0.63 |
| PS | PS (36:1) | $C_{42}H_{80}NO_{10}P$ | $[M-H]^-$ | 788.5447 | 788.5456 | 1.12 | 152, 227, 701, 788 | C | 0.75 |
| PS | PS (36:2) | $C_{42}H_{78}NO_{10}P$ | $[M-H]^-$ | 786.5291 | 786.5298 | 0.85 | 152, 255, 699, 786 | W, C, OM | 0.61 |
| PS | PS (36:4) | $C_{42}H_{74}NO_{10}P$ | $[M-H]^-$ | 782.4978 | 782.4987 | 1.18 | 227, 695, 782 | W, C | 0.65 |
| PS | PS (40:6) | $C_{46}H_{78}NO_{10}P$ | $[M-H]^-$ | 834.5291 | 834.5322 | 3.77 | 303, 834 | W, C, OM, IM | 0.39 |
| Others | 3-O-Sulfogalactosylceramide (d18:1/24:0) | $C_{48}H_{93}NO_{11}S$ | $[M-H]^-$ | 890.6397 | 890.6391 | -0.67 | 890 | OM | 0.57 |
| Others | Glucose | $C_6H_{12}O_6$ | $[M-H]^-$ | 179.0561 | 179.0563 | 1.06 | 161, 179 | C, OM | 1.27 |
| Others | Chlorphenesin | $C_9H_{11}ClO_3$ | $[M-H]^-$ | 201.0324 | 201.0320 | -1.94 | 59 | W, C, OM, IM | 0.31 |
| Others | Ajoene | $C_9H_{14}OS_3$ | $[M-H]^-$ | 233.0134 | 233.0138 | 1.85 | 78, 233 | OM, IM | 0.57 |
| Others | 3-Hydroxysintaxanthin | $C_{31}H_{42}O_2$ | $[M-H]^-$ | 445.3112 | 445.3118 | 1.24 | 445 | W, C, OM, IM | 3.18 |
| Others | Cholesterol sulfate | $C_{27}H_{46}O_4S$ | $[M-H]^-$ | 465.3044 | 465.3050 | 1.19 | 465 | W, C, OM, IM | 0.50 |

DG: diacylglycerol; FAHFA: fatty acid esters of hydroxy fatty acids; LysoPE: lysophosphatidylethanolamine; PA: phosphatidic acid; LysoPG: lysophosphatidylglycerol; LysoPI: lysophosphatidylinositol; PE: phosphatidylethanolamine; PG: phosphatidylglycerol; PI: Phosphatidylinositol; PS: phosphatidylserine, AMP: adenosine monophosphate. W: whole kidney; C: cortex; OM: outer medulla; IM: inner medulla.

**Table S3**. Discriminating metabolites obtained by positive AFADESI-MSI analysis of renal sections from control and HFD/STZ-induced diabetic nephropathy (DN) group.

| Species | Metabolite identification | Elemental composition | Adduct | Theoretical *m/z* | Measured *m/z* | Delta (ppm) | MS/MS | Distribution | FC(W) |
|---|---|---|---|---|---|---|---|---|---|
| Choline | Choline | $C_5H_{13}NO$ | $[M+H]^+$ | 104.1070 | 104.1071 | 0.76 | 58, 60, 104 | W, C, OM, IM | 0.54 |
| Carnitine | L-Carnitine | $C_7H_{15}NO_3$ | $[M+H]^+$ | 162.1122 | 162.1125 | 1.79 | 60, 85, 102, 103, 144, 162 | W, C, IM | 0.51 |
| Carnitine | Stearoylcarnitine | $C_{25}H_{39}NO_4$ | $[M+H]^+$ | 428.3734 | 428.3728 | -1.50 | 85, 369, 428; | W, C, IM | 3.39 |
| PC | PC (34:1) | $C_{42}H_{82}NO_8P$ | $[M+H]^+$ | 760.5851 | 760.5842 | -1.19 | 86, 104, 124b, 184, 760 | IM | 1.47 |
| PC | PC (34:2) | $C_{42}H_{80}NO_8P$ | $[M+H]^+$ | 758.5694 | 758.5694 | 0.01 | 86, 124b, 184, 758 | C | 0.64 |
| PC | PC (36:1) | $C_{44}H_{86}NO_8P$ | $[M+Na]^+$ | 810.5983 | 810.5978 | -0.56 | 184, 146 | OM | 1.64 |
| PC | PC (36:2) | $C_{44}H_{84}NO_8P$ | $[M+K]^+$ | 824.5566 | 824.5554 | -1.48 | 104, 184 | OM | 1.29 |
| PC | PC (36:4) | $C_{44}H_{80}NO_8P$ | $[M+Na]^+$ | 804.5514 | 804.5478 | -4.50 | 184 | C | 1.20 |
| PE | PE (p-18:0/20:4 (5Z,8Z,11Z, 14Z)) | $C_{43}H_{78}NO_7P$ | $[M+Na]^+$ | 774.5408 | 774.5401 | -0.87 | - | W, C, OM | 1.90 |
| SM | SM (d18:1/16:0) | $C_{39}H_{79}N_2O_6P$ | $[M+Na]^+$ | 725.5568 | 725.5556 | -1.69 | 502, 542b, 666, 725 | W, C | 2.91 |
| SM | SM (42:2) | $C_{47}H_{93}N_2O_6P$ | $[M+H]^+$ | 813.6844 | 813.6828 | -1.93 | - | OM | 0.89 |
| Others | Creatine | $C_4H_9N_3O_2$ | $[M+H]^+$ | 132.0768 | 132.0768 | 0.30 | 90, 115, 132 | W, C | 0.15 |

PC: phosphatidylcholine; PE: phosphatidylethanolamine; SM: sphingomyelin. W: whole kidney; C: cortex; OM: outer medulla; IM: inner medulla.

**Table S4**. Discriminating metabolites obtained by negative AFADESI-MSI analysis of renal sections from control and db/db diabetic nephropathy (DN) group.

| Species | Metabolite identification | Elemental composition | Adduct | Theoretical *m/z* | Measured *m/z* | Delta (ppm) | MS/MS | Distribution | FC(W) |
|---|---|---|---|---|---|---|---|---|---|
| Amino- acid | Glutamate | $C_5H_9NO_4$ | $[M-H]^-$ | 146.0459 | 146.0452 | -4.54 | 85, 100, 102, 128, 129, 146 | W, C, OM, IM | 0.72 |
| Amino- acid | Histidylaspartic acid | $C_{10}H_{14}N_4O_5$ | $[M-H]^-$ | 269.0891 | 269.0892 | 0.39 | 59b, 269 | W, C, OM, IM | 14.77 |
| Fatty- acid | Palmitelaidic acid | $C_{16}H_{30}O_2$ | $[M-H]^-$ | 253.2173 | 253.2178 | 1.78 | 253 | OM, IM | 0.79 |
| Fatty- acid | Linolenic acid | $C_{18}H_{30}O_2$ | $[M-H]^-$ | 277.2173 | 277.2181 | 3.01 | 59, 277 | W, C | 0.77 |
| Fatty- acid | Eicosenoic acid | $C_{20}H_{38}O_2$ | $[M-H]^-$ | 309.2799 | 309.2794 | -1.46 | 59, 309 | W, C, OM, IM | 0.65 |
| Fatty- acid | Arachidonic acid | $C_{20}H_{32}O_2$ | $[M-H]^-$ | 303.233 | 303.2338 | 2.48 | 59, 259, 303 | OM | 0.90 |
| Fatty- acid | Eicosapentaenoic acid | $C_{20}H_{30}O_2$ | $[M-H]^-$ | 301.2173 | 301.2183 | 3.38 | 59, 257, 301 | OM | 1.21 |
| LysoPG | LysoPG (18:1) | $C_{24}H_{47}O_9P$ | $[M-H]^-$ | 509.2885 | 509.2896 | 2.26 | 152, 227, 281, 509 | W, OM, IM | 2.09 |
| LysoPG | LysoPG (18:2) | $C_{24}H_{45}O_9P$ | $[M-H]^-$ | 507.2728 | 507.2745 | 3.38 | 152, 227, 245, 279, 415, 433, 507 | W, C, OM, IM | 1.73 |
| Nucleoside& Nucleotide& Nitrogenbase | AMP | $C_{10}H_{14}N_5O_7P$ | $[M-H]^-$ | 346.0558 | 346.0565 | 2.13 | 78, 96, 134, 211, 346 | W, C, OM, IM | 1.88 |
| Nucleoside& Nucleotide& Nitrogenbase | GMP | $C_{10}H_{14}N_5O_8P$ | $[M-H]^-$ | 362.0507 | 362.0515 | 2.19 | 78, 96, 150, 362 | OM | 1.27 |
| Organic-acid | Succinate | $C_4H_6O_4$ | $[M-H]^-$ | 117.0193 | 117.0195 | 1.47 | 71, 73, 117 | W, C, OM | 1.92 |
| Organic-acid | Malate | $C_4H_6O_5$ | $[M-H]^-$ | 133.0142 | 133.0147 | 3.97 | 71, 72, 89, 115, 133 | OM | 0.83 |
| Organic-acid | dobesilic acid | $C_6H_6O_5S$ | $[M-H]^-$ | 188.9863 | 188.9865 | 1.18 | 80, 188 | W, C, OM, IM | 7.46 |
| PA | PA (16:0/18:1(9Z)) | $C_{37}H_{71}O_8P$ | $[M-H]^-$ | 673.4814 | 673.4801 | -1.97 | 255, 673 | W, C, OM, IM | 3.32 |
| PA | PA (38:4) | $C_{41}H_{72}O_8P$ | $[M-H]^-$ | 723.49698 | 723.4943 | -3.74 | 152, 281, 723 | W, C, OM | 1.39 |
| PA | PA (41:5) | $C_{44}H_{75}O_9P$ | $[M-H]^-$ | 777.5076 | 777.5060 | -2.01 | 777 | W, C, OM, IM | 0.06 |

| | | | | | | | | | |
|---|---|---|---|---|---|---|---|---|---|
| PC | PC (36:4) | $C_{44}H_{80}NO_8P$ | $[M+Cl]^-$ | 816.5316 | 816.5328 | 1.43 | 209, 211 | W, C, OM | 1.54 |
| PE | PE (p-34:1) | $C_{39}H_{76}NO_7P$ | $[M-H]^-$ | 700.5287 | 700.5292 | 0.73 | 700 | OM | 1.40 |
| PE | PE (34:1) | $C_{39}H_{76}NO_8P$ | $[M-H]^-$ | 716.5235 | 716.5253 | 2.46 | 716 | OM | 1.04 |
| PE | PE (36:4) | $C_{41}H_{74}NO_8P$ | $[M-H]^-$ | 738.5079 | 738.5103 | 3.27 | 738 | C | 1.29 |
| PE | PE (36:6) | $C_{41}H_{70}NO_8P$ | $[M-H]^-$ | 734.4766 | 734.4763 | -0.47 | 734 | OM | 0.98 |
| PE | PE (p-38:4) | $C_{43}H_{78}NO_7P$ | $[M-H]^-$ | 750.5443 | 750.5451 | 1.08 | 750 | C | 1.30 |
| PE | PE (40:1) | $C_{45}H_{86}NO_9P$ | $[M+Cl]^-$ | 850.5734 | 850.5738 | 0.48 | - | IM | 1.47 |
| PE | PE (p-40:4) | $C_{45}H_{82}NO_7P$ | $[M-H]^-$ | 778.5756 | 778.5777 | 2.64 | 778 | W, OM | 0.53 |
| PE | PE (p-40:7) | $C_{45}H_{76}NO_7P$ | $[M-H]^-$ | 772.5287 | 772.5294 | 0.85 | 327, 444, 772 | W, C | 0.74 |
| PE | PE (42:1) | $C_{46}H_{89}NO_{12}S$ | $[M-H]^-$ | 878.6032 | 878.6052 | 2.23 | - | W, C, IM | 1.58 |
| PG | PG (32:0) | $C_{38}H_{75}O_{10}P$ | $[M-H]^-$ | 721.5025 | 721.5041 | 2.28 | 152, 255, 721 | W, C, OM | 0.28 |
| PG | PG (34:1) | $C_{40}H_{77}O_{10}P$ | $[M-H]^-$ | 747.5182 | 747.5187 | 0.62 | 152, 171, 255, 281, 465, 747 | W, C, OM, IM | 1.55 |
| PG | PG (38:4) | $C_{44}H_{79}O_{10}P$ | $[M-H]^-$ | 797.5338 | 797.5342 | 0.45 | 279, 307, 507, 797 | C | 0.64 |
| PG | PG (40:8) | $C_{46}H_{75}O_{10}P$ | $[M-H]^-$ | 817.5025 | 817.5056 | 3.77 | 817 | IM | 0.90 |
| PI | PI (34:0) | $C_{43}H_{83}O_{13}P$ | $[M-H]^-$ | 837.5499 | 837.5508 | 2.20 | 283, 419, 837 | OM | 1.29 |
| PI | PI (38:4) | $C_{47}H_{83}O_{13}P$ | $[M-H]^-$ | 885.5499 | 885.5519 | 2.22 | 419, 303, 439, 581, 599, 885 | W, C, IM | 1.40 |
| PI | PI (38:5) | $C_{47}H_{81}O_{13}P$ | $[M-H]^-$ | 883.5342 | 883.5361 | 2.19 | 303, 883 | W, C, IM | 1.47 |
| PI | PI (40:6) | $C_{49}H_{83}O_{13}P$ | $[M-H]^-$ | 909.5499 | 909.5522 | 2.48 | 283, 419, 625, 909 | W, C, OM | 0.60 |
| PS | PS (36:1) | $C_{42}H_{80}NO_{10}P$ | $[M-H]^-$ | 788.5447 | 788.5417 | -3.78 | 152, 701, 788 | IM | 1.34 |
| PS | PS (36:4) | $C_{42}H_{74}NO_{10}P$ | $[M-H]^-$ | 782.4978 | 782.4998 | 2.58 | 695, 782 | W, C | 1.53 |
| Others | Glucose | $C_6H_{12}O_6$ | $[M+Cl]^-$ | 215.0328 | 215.0325 | -1.21 | 59, 71, 87, 119 | W, C, OM, IM | 5.97 |
| Others | Prinomide | $C_{15}H_{13}N_3O_2$ | $[M+Cl]^-$ | 302.0702 | 302.0701 | -0.22 | - | W, C, OM, IM | 0.51 |
| Others | GSH | $C_{10}H_{17}N_3O_6S$ | $[M-H]^-$ | 306.0766 | 306.0763 | -0.99 | 59, 127 | W, C, OM | 1.85 |
| Others | Calycanthidine | $C_{23}H_{28}N_4$ | $[M-H]^-$ | 359.2241 | 359.2235 | -1.54 | 359 | W, C, OM | 0.27 |
| Others | 3-O-Sulfogalactosylceramide (d18:1/24:0) | $C_{48}H_{93}NO_{11}S$ | $[M-H]^-$ | 890.6397 | 890.6414 | 1.85 | 890 | C | 0.94 |

LysoPG: lysophosphatidylglycerol; PA: phosphatidic acid; PC: phosphatidylcholine; PE: phosphatidylethanolamine; PG: phosphatidylglycerol; PI: Phosphatidylinositol; PS: phosphatidylserine. W: whole kidney; C: cortex; OM: outer medulla; IM: inner medulla.

**Table S5**. Discriminating metabolites obtained by positive AFADESI-MSI analysis of renal sections from control and db/db diabetic nephropathy (DN) group.

| Species | Metabolite identification | Elemental composition | Adduct | Theoretical *m/z* | Measured *m/z* | Delta (ppm) | MS/MS | Distribution | FC(W) |
|---|---|---|---|---|---|---|---|---|---|
| Choline | Choline | $C_5H_{13}NO$ | $[M+H]^+$ | 104.1070 | 104.1070 | 0.39 | 58, 60, 86, 104 | W, OM, IM | 0.62 |
| Choline | GPC | $C_8H_{20}NO_6P$ | $[M+K]^+$ | 296.0660 | 296.0656 | -1.48 | 84, 86, 104 | C, IM | 1.13 |
| Carnitine | Palmitoylcarnitine | $C_{23}H_{45}NO_4$ | $[M+H]^+$ | 400.3421 | 400.3427 | 1.51 | 85, 341, 400 | W, OM, IM | 1.74 |
| Carnitine | Linoleylcarnitine | $C_{25}H_{45}NO_4$ | $[M+H]^+$ | 424.3421 | 424.3434 | 3.17 | 85, 424 | IM | 3.19 |
| Carnitine | Octadecenoylcarnitine | $C_{25}H_{47}NO_4$ | $[M+H]^+$ | 426.3578 | 426.3585 | 1.56 | 85, 426 | W, OM, IM | 3.28 |
| LysoPC | LysoPC (18:0) | $C_{26}H_{54}NO_7P$ | $[M+H]^+$ | 524.3711 | 524.3708 | -0.49 | 86, 104, 124, 184, 240, 258, 341, 506, 524 | W, C, OM, IM | 2.70 |
| PC | PC (34:0) | $C_{42}H_{84}NO_8P$ | $[M+H]^+$ | 762.6007 | 762.6020 | 1.74 | 86, 184, 762 | W, C, OM, IM | 2.51 |
| PC | PC (34:1) | $C_{42}H_{82}NO_8P$ | $[M+H]^+$ | 760.5851 | 760.5870 | 2.51 | 124, 184, 760 | W, C, OM, IM | 1.90 |
| PC | PC (34:2) | $C_{42}H_{80}NO_8P$ | $[M+H]^+$ | 758.5694 | 758.5711 | 2.22 | 184 | OM | 1.16 |
| PC | PC (36:2) | $C_{44}H_{84}NO_8P$ | $[M+H]^+$ | 786.6007 | 786.6018 | 1.36 | 124, 184, 603, 727, 786 | W, OM, IM | 1.57 |
| PC | PC (36:3) | $C_{44}H_{82}NO_8P$ | $[M+Na]^+$ | 806.5670 | 806.5659 | -1.41 | 184, 623, 747, 806 | C, OM, IM | 1.13 |

| | | | | | | | | | |
|---|---|---|---|---|---|---|---|---|---|
| PC | PC (38:4) | $C_{46}H_{84}NO_8P$ | $[M+Na]^+$ | 832.5827 | 832.5801 | -3.09 | 184, 649, 773, 832 | IM | 1.01 |
| PC | PC(p-38:5) | $C_{46}H_{82}NO_7P$ | $[M+Na]^+$ | 814.5721 | 814.5718 | -0.31 | 184 | W, C, OM, IM | 0.42 |
| PE | PE(p-36:4) | $C_{41}H_{74}NO_7P$ | $[M+H]^+$ | 724.5276 | 724.5274 | -0.21 | 724 | W, OM | 1.99 |
| SM | SM(d18:1/16:0) | $C_{39}H_{79}N_2O_6P$ | $[M+K]^+$ | 741.5307 | 741.5313 | 0.83 | 184, 666 | OM | 1.07 |
| SM | SM(d18:1/24:1(15Z)) | $C_{47}H_{93}N_2O_6P$ | $[M+K]^+$ | 851.6403 | 851.6398 | -0.57 | - | OM | 1.14 |
| Others | betaine | $C_5H_{11}NO_2$ | $[M+H]^+$ | 118.0862 | 118.0860 | -1.86 | 58, 59, 74, 100, 118 | W, C | 1.78 |

GPC: Glycerophosphocholine; LysoPC: lysophosphatidylcholine; PC: phosphatidylcholine; PE: phosphatidylethanolamine; SM: sphingomyelin. W: whole kidney; C: cortex; OM: outer medulla; IM: inner medulla.

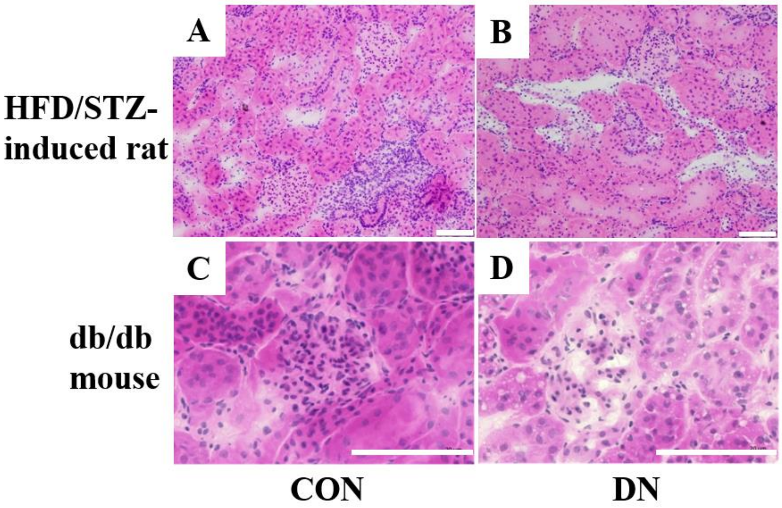


**Figure S1.** H&E staining in the HFD/STZ-induced diabetic rats (**A, B**) and db/db mice (**C, D**). CON: control group; DN: diabetic nephropathy group; HFD/STZ-induced rat: High-Fat diet feeding combined with intraperitoneal injection of low dose of STZ. Scale bar: 100um.

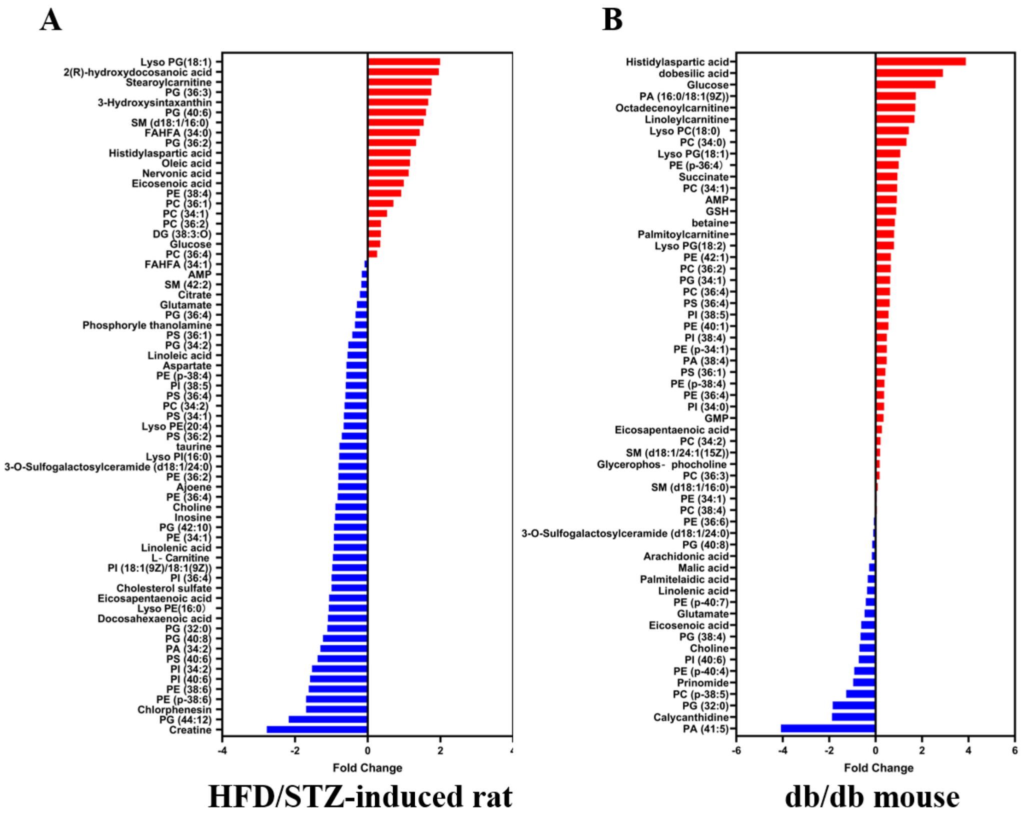


**Figure S2.** Fold change plots of discriminating metabolites in HFD/STZ-induced diabetic rats versus db/db mice. PC: phosphatidylcholine; PE: phosphatidylethanolamine; PS: phosphatidylserine; PI: phosphatidylinositol; PG: phosphatidylglycerol; DG: diacylglycerol; SM: sphingomyelin; LysoPG: lysophosphatidylglycerol; LysoPI: lysophosphatidylinositol; PA: phosphatidic acid; LysoPC: Lysophosphatidylcholine; FAHFAs: fatty acid esters of hydroxy fatty acids.

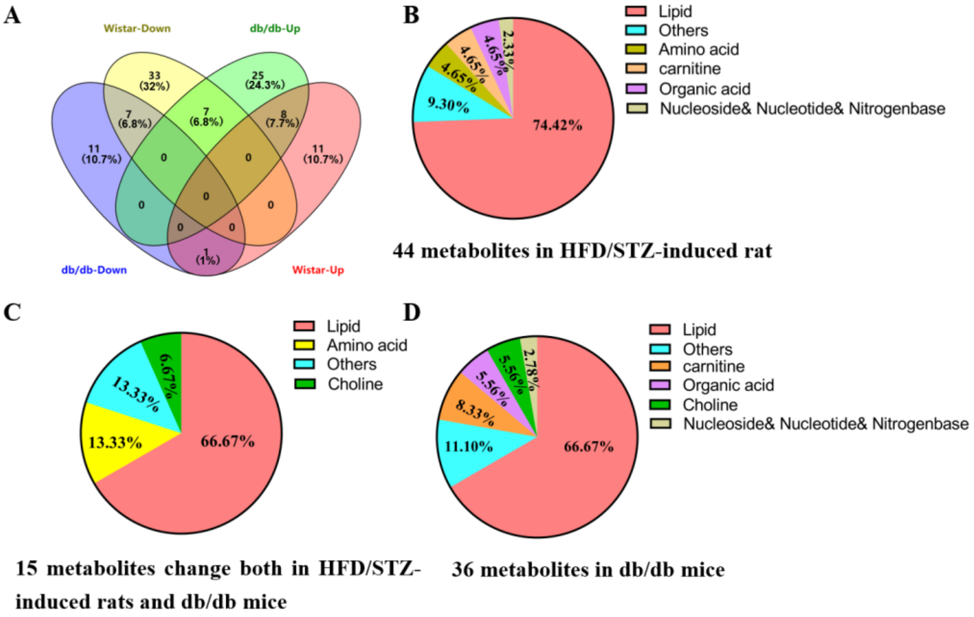


**Figure S3.** The comparison of discriminating metabolites associated DN between the two models. Wistar-down: Wistar rats discriminating metabolites were down-regulated in the HFD/STZ-induced diabetic rats compared to the control group. Wistar-up: Wistar rats discriminating metabolites were up-regulated in the HFD/STZ-induced diabetic rats compared to the control group. db/db-down: db/db mice discriminating metabolites were down-regulated compared to the control group. db/db-up: db/db mice discriminating metabolites were up-regulated compared to the control group.

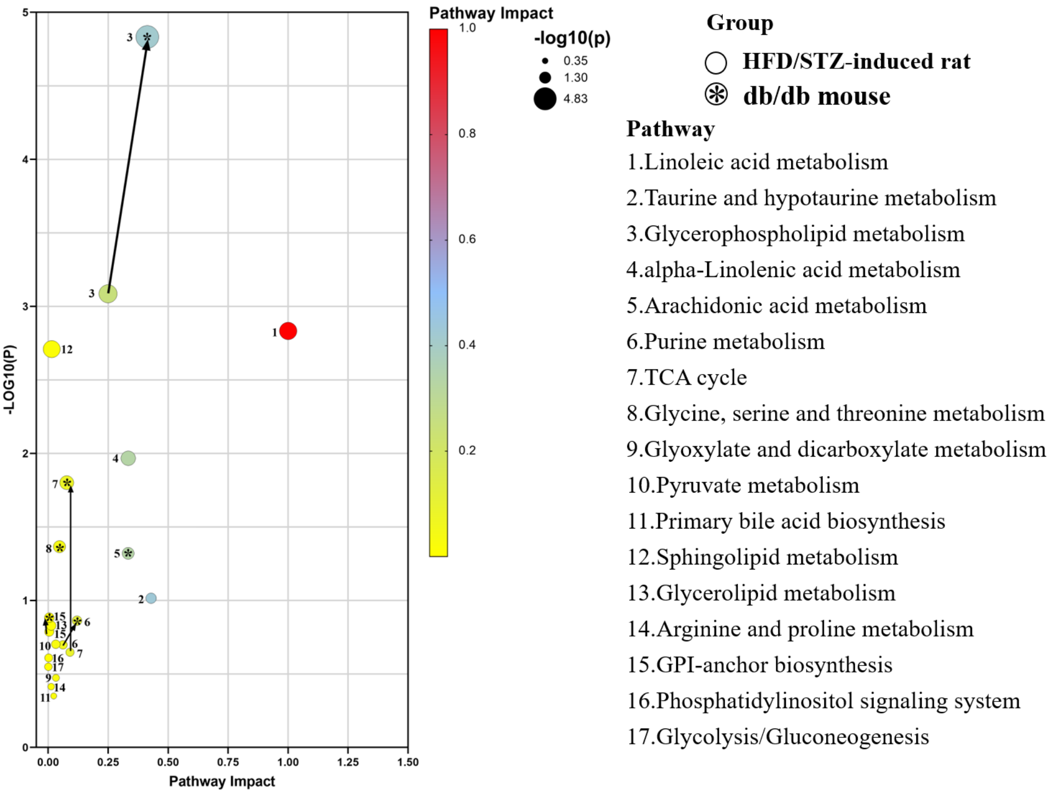


**Figure S4.** Regulation of discriminating metabolites in HFD/STZ-induced diabetic rats and db/db mice. HFD: High-Fat diet. STZ: Streptozotocin. TCA: tricarboxylic acid. GPI-anchor biosynthesis: Glycosylphosphatidylinositol-anchor biosynthesis metabolism.

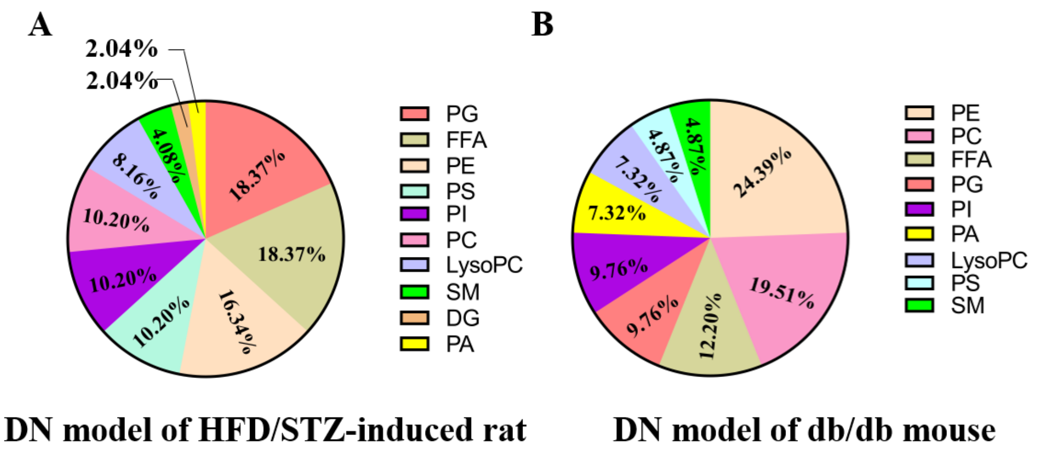


**Figure S5.** Fan chart of discriminating lipid species in HFD/STZ-induced diabetic rats

and db/db mice. PG: phosphatidylglycerol; FFA: free fatty acid; PC: phosphatidylcholine; PE: phosphatidylethanolamine; PS: phosphatidylserine; PI: phosphatidylinositol; DG: diacylglycerol; SM: sphingomyelin; LysoPG: lysophosphatidylglycerol; LysoPI: lysophosphatidylinositol; PA:phosphatidic acid; LysoPC: Lysophosphatidylcholine; FAHFAs: fatty acid esters of hydroxy fatty acids.

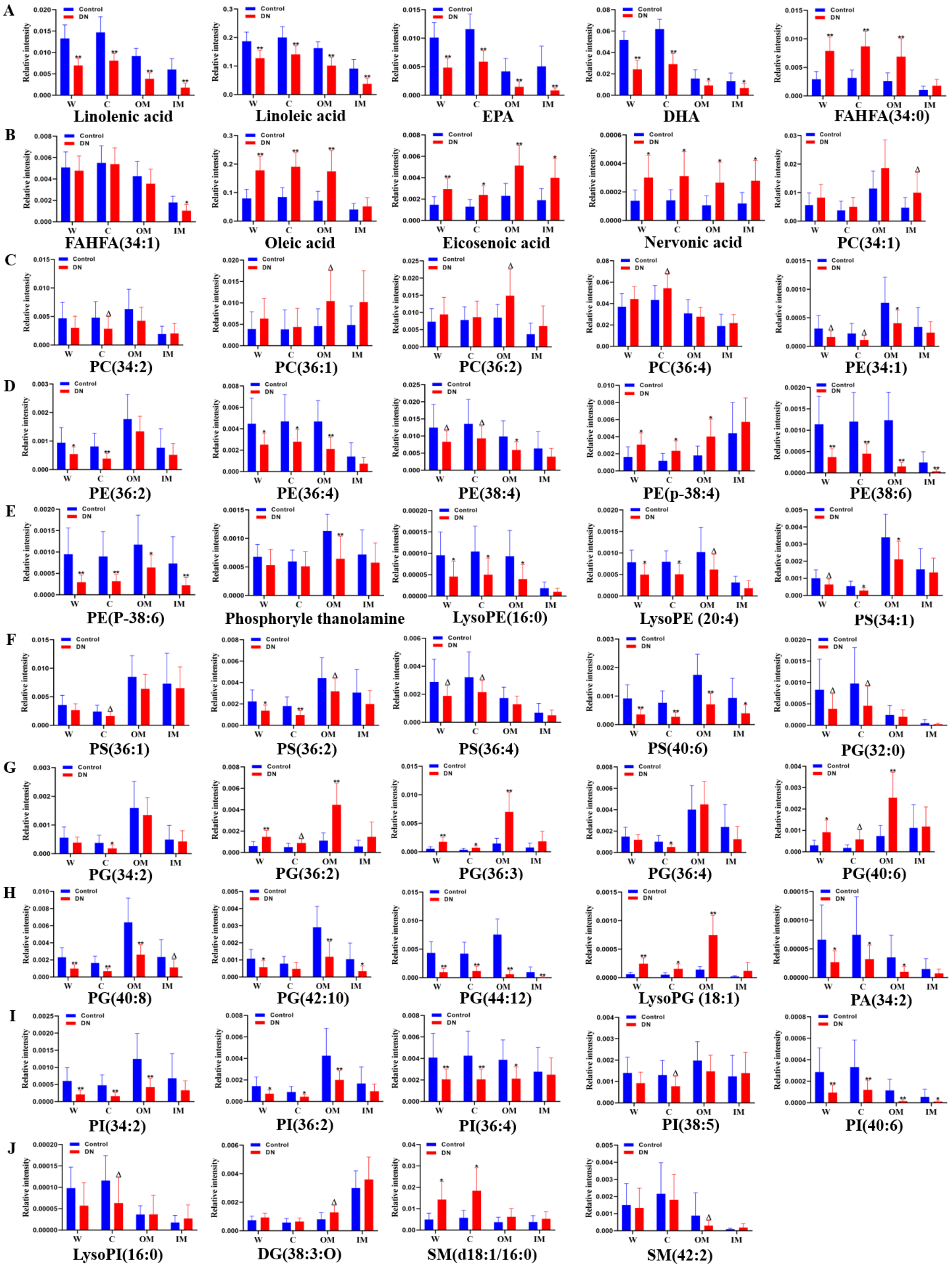

**Figure S6.** Histogram of metabolites involved in lipid metabolism in HFD/STZ-induced diabetic rats. W: whole; C: cortex; OM: outer medullar; IM: inner medullar. Control: control group; DN: diabetic nephropathy groups; EPA: eicosapentaenoic acid; DHA: do-cosahexaenoic acid; FAHFAs: fatty acid esters of hydroxy fatty acids; PC: phosphatidylcholine; PE:phosphatidylethanolamine; PE(38:4):PE(P18:0/20:4(5Z,8Z,11Z,14Z)); LysoPE: lysophosphatidylethanolamine; PS: phosphatidylserine; PG: phosp-hatidylglycerol; LysoPG: lysophosphatidylglycerol; PA: phosphatidic acid; PI: phosph-atidylinositol; LysoPI: lysophosphatidylinositol; PI(36:2): PI(18:1(9Z):18:1(9Z)); DG: diacylglycerol; SM: sphingomyelin. Scale bar: 3mm, $^{\triangle}P < 0.1$, $^{*}P < 0.05$, $^{**}P < 0.01$.

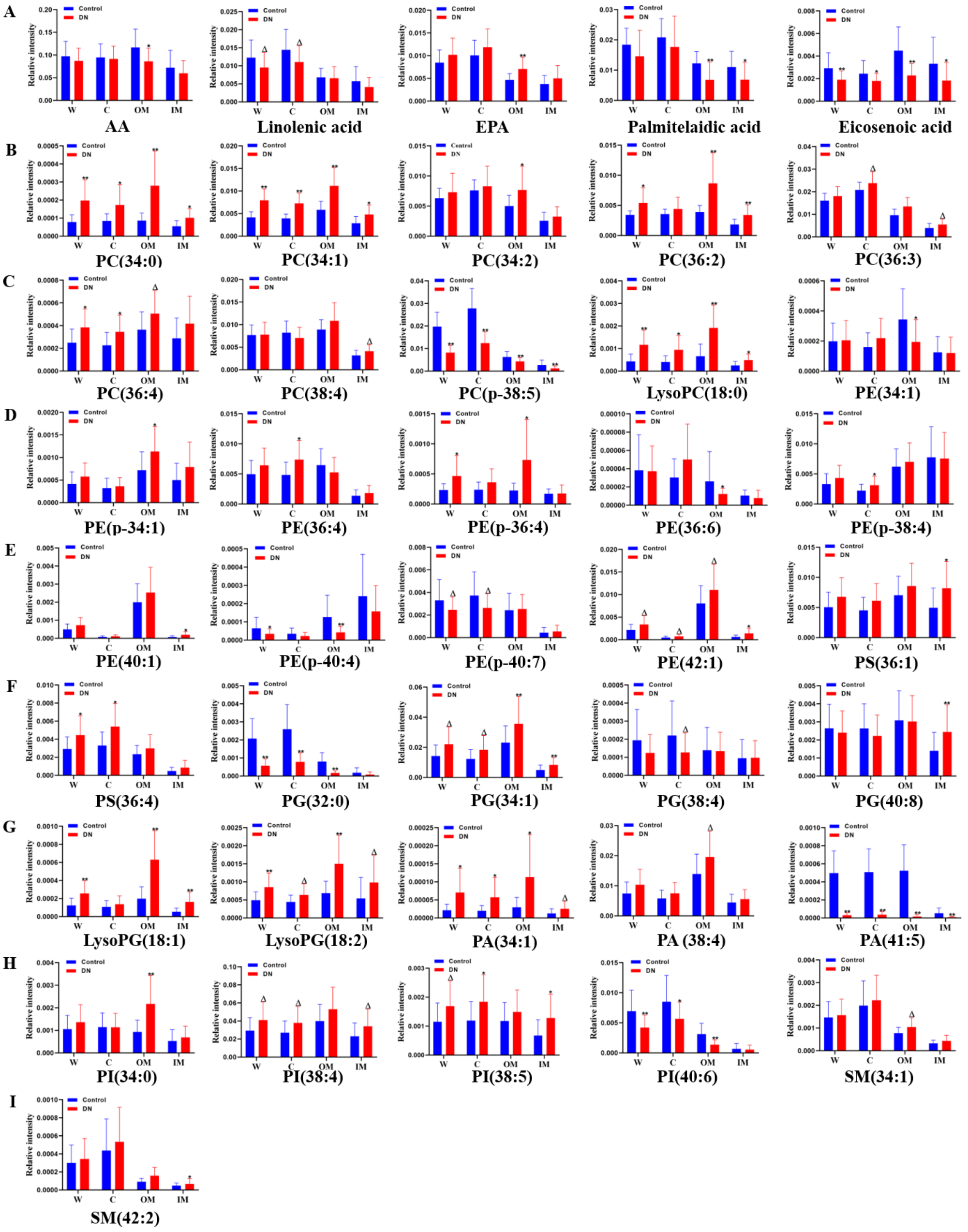


Figure S7. Histogram of metabolites involved in lipid metabolism in db/db mice. W: whole; C: cortex; OM: outer medullar; IM: inner medullar. Control: control group; DN: diabetic nephropathy groups; AA: Arachidonic acid; EPA: eicosapentaenoic acid; PC: phosphatidylcholine; LysoPC: Lysophosphatidylcholine; PE: phosphatidylethanolamine; PS: phosphatidylserine; PG: phosphatidylglycerol; LysoPG: lysophosphatidylgly-

cerol; PA: phosphatidic acid; PA(34:1): 16:0/18:1(9Z)); PI: phosphatidylinositol; SM: sphingomyelin; SM (34:1): SM(d18:1/16:0); SM(42:2): SM(d18:1/24:1(15Z)); Scale bar: 3mm, $^{\triangle}P < 0.1$, $^{*}P < 0.05$, $^{**}P < 0.01$.

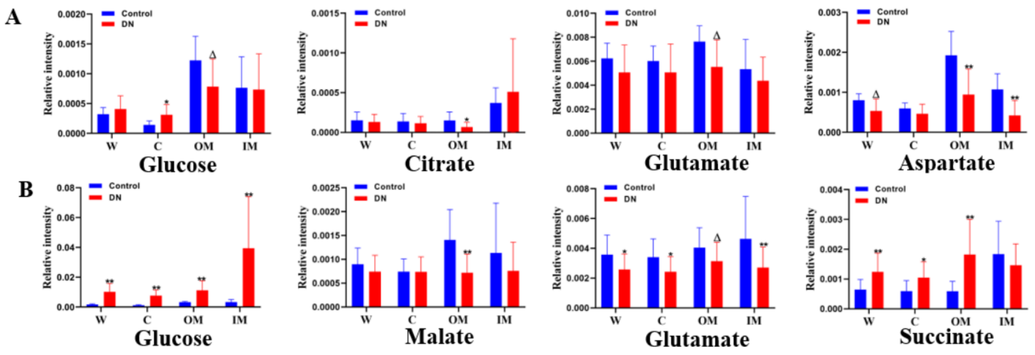


Figure S8. Histogram of metabolites involved in glycolysis and TCA cycle in HFD/STZ-induced diabetic rats (A) and db/db mice(B). W: whole; C: cortex; OM: outer medullar; IM: inner medullar. Control: control group; DN: diabetic nephropathy groups; Scale bar: 3mm, $^{\triangle}P < 0.1$, $^{*}P < 0.05$, $^{**}P < 0.01$.

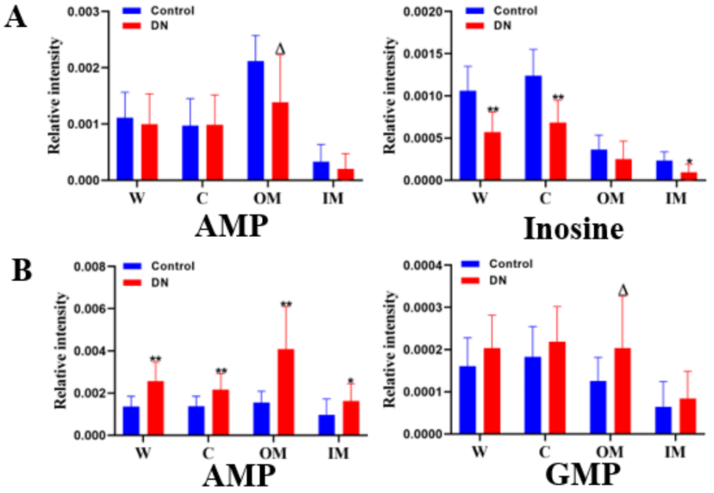


Figure S9. Histogram of metabolites involved in purine metabolism in HFD/STZ-induced diabetic rats (A) and db/db mice(B). W: whole; C: cortex; OM: outer medullar;

IM: inner medullar. Control: control group; DN: diabetic nephropathy groups; AMP: adenosine monophosphate; GMP: guanine monophosphate. Scale bar: 3mm, $^{\triangle}P < 0.1$, $^{*}P < 0.05$, $^{**}P < 0.01$.

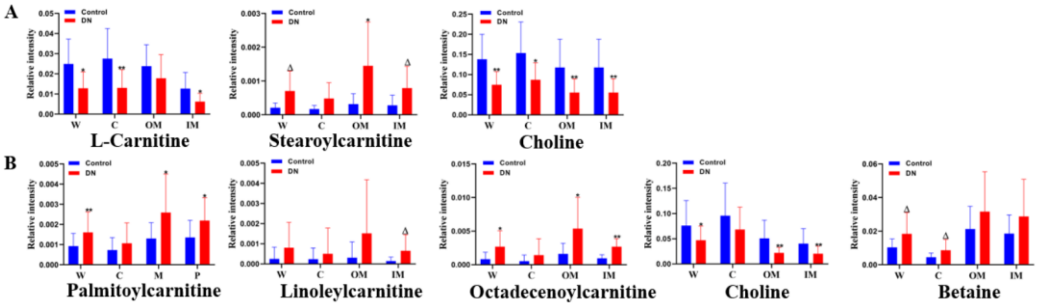


Figure S10. Histogram of metabolites involved in carnitine and choline metabolism in HFD/STZ-induced diabetic rats (A) and db/db mice(B). W: whole; C: cortex; OM: outer medullar; IM: inner medullar. Control: control group; DN: diabetic nephropathy groups; Scale bar: 3mm, $^{\triangle}P < 0.1$, $^{*}P < 0.05$, $^{**}P < 0.01$.

## Supplementary Methods

### 1. LC-MS/MS analysis

LC-MS/MS experiments were performed in positive and negative ion mode on a Q-OT-qIT hybrid mass spectrometer (Orbitrap Fusion Lumos, Thermo Fisher Scientific, USA). Renal tissue samples were weighed at approximately 100 mg and placed into 2 ml centrifuge tubes, homogenized in a ratio of 1 g: 4 mL in 0.4 mL methanol/water (8:2 v/v). Sonicate the sample for 10 minutes, the supernatant was obtained by centrifugation at 12,000 R/min for 10 min at 4 °C. The supernatant was then concentrated in a centrifugal vacuum concentrator (CHRIST RVC 2-25 CD plus,

CHRIST, Osterode, Germany) for 4 h. The residues were dissolved in 200 uL of 1% acetonitrile and centrifugation was continued for 10 min under the same conditions. Then the supernatant was transfered to a sample vial for LC-MS/MS analysis. For the ions of interest, targeting analysis was performed in full scan and $DDMS^2$ scan modes with normalized HCD collision energy values set to 15%, 30% and 45%. For MS/MS acquisition, the mass resolving was set to 15000, with a maximum injection time of 100 ms and a range of 67–1000 Da.
